# Serum folate concentration and the incidence of lung cancer

**DOI:** 10.1371/journal.pone.0177441

**Published:** 2017-05-11

**Authors:** Katarzyna Durda, Krzysztof Kąklewski, Satish Gupta, Michał Szydłowski, Piotr Baszuk, Katarzyna Jaworska-Bieniek, Grzegorz Sukiennicki, Katarzyna Kaczmarek, Piotr Waloszczyk, Steven Narod, Jan Lubiński, Anna Jakubowska

**Affiliations:** 1 Departmentof Genetics and Pathology, International Hereditary Cancer Center, Pomeranian Medical University, Połabska 4, Szczecin, Poland; 2 Faculty of Mechanical Engineering and Mechatronics, West Pomeranian University of Technology Szczecin, al. Piastów 19, Szczecin, Poland; 3 Independent Laboratory of Pathology, Zdunomed, Szczecin, Poland; 4 Womens College Research Institute, Toronto, Ontario, Canada; National Health Research Institutes, TAIWAN

## Abstract

**Background:**

Lung cancer is a leading cause of cancer-related mortality globally. Folate helps to maintain DNA integrity and to regulate gene expression. Serum folate levels may affect the risk of several cancers, including lung cancer. In this study we evaluated the association between serum folate concentration and variations in genes involved in folate metabolism with lung cancer incidence in Poland.

**Methods:**

The study included 366 lung cancer patients and 366 control subjects. We measured serum folate concentration and genotyped six variants in *MTHFR*, *MTR* and *MTRR* genes. The odds ratios of being diagnosed with lung cancer were calculated using conditional univariable and multivariable logistic regression with respect to folate level and genotypes.

**Results:**

The mean serum folate level was lower in lung cancer cases than in control group (20.07 nmol/l vs. 22.52 nmol/l, p = 0.002). The odds ratio for lung cancer declined with increasing serum content of the folate. The folate concentration of >25.71 nmol/l (IVth quartile) in comparison to <15.92 nmol/l (Ist quartile) was associated with an odds ratio of 0.61 (95%CI 0.40–0.95, p = 0.03). The analysis of variations in *MTHFR*, *MTR* and *MTRR* genes did not reveal any significant difference between lung cancer cases and controls in univariable and multivariable analyses.

**Conclusion:**

In this case-control study, lower serum folate concentrations were associated with a higher risk of lung cancer diagnosis. Although previous findings have been somewhat mixed, our results add to the evidence that circulating folate levels may be an indicator of lung cancer risk.

## Introduction

Lung cancer is the most common cancer and a leading cause of cancer-related mortality globally [1]. In 2013 in Poland, 22,600 deaths from lung cancer were recorded (http://85.128.14.124/krn/). Tobacco smoke is responsible for the majority of cases, however 10–20% patients develop the disease without a history of tobacco use [2, 3].

The pathogenesis of lung cancer involves the accumulation of genetic and epigenetic alterations resulting in impairment of function of key oncogenes, tumor suppressor genes, and DNA repair genes [3, 4]. DNA methylation is an epigenetic modification that is critical for gene regulation and development. In tumors it may result in the silencing of tumor suppressor genes and chromosome instability [5]. Aberrant methylation, including gene-specific hyper-methylation, has also been indicated as an early event in carcinogenesis and progression of lung cancer [6].

Folates play an essential role in one-carbon transfer involving remethylation of homocysteine to methionine, which is a precursor of S-adenosylmethionine, the primary methyl group donor for most biological methylations, including DNA. Folate deficiency is associated with altered DNA methylation and synthesis, and disruption of DNA repair activities, which may be an underlying molecular mechanism for the association between folate concentration and cancer risk [7]. Several studies have examined an association of folate status and lung cancer, but results were inconclusive [8–19]. These studies differed between in method of folate status determination (dietary intake or serum/plasma concentration), and did not account for potential effect of genetic factors. Folate metabolism is influenced not only by its dietary intake and absorption but also is regulated by several enzymes [20]. The methylenetetrahydrofolate reductase (MTHFR), methionine synthase (MTR) and methionine synthase reductase (MTRR) are important enzymes involved in DNA synthesis and the generation of S-adenosylmethionine (SAM)—a universal methyl-donor for methylation reactions [21]. Several reports suggested that genetic polymorphisms in these genes can be associated with lung cancer risk [22–25], which may be modulated by folate intake [26, 27].

In this study, we aimed to evaluate the association of serum folate concentration and of six known functional variations in *MTHFR*, *MTR*, *MTRR* genes and the risk of lung cancer in Poland.

## Materials and methods

### Study participants

The study group consisted of 366 consecutive patients with lung cancer diagnosed at Clinical Thoracic Surgery Department in Szczecin between January 2013 and January 2016. All lung cancers were histologically confirmed. Clinical details are presented in Table 1. The patients with any malignancy diagnosed in the past were not included in the study. The blood samples from all cases were collected at the time of lung cancer diagnosis, but before treatment. The controls consisted of 366 healthy individuals selected from our registry, which was complementary biorepository constructed as part of a population-based study of the 1.7 million inhabitants of West Pomerania designed to identify familial aggregations of cancer performed by our center. For each diagnosed lung cancer case, several cancer–free controls matched for sex and year of birth (+/−3 years) were invited for interview. This allowed additional matching for total number of lung and other cancers first degree relatives, as well as smoking history (pack-years), in the final selection of one control per case. Control individuals meeting matching criteria were identified by a review of the records of the population based study and invited for an interview and blood donation. This was performed simultaneously with recruitment of lung cancer cases. The characteristic of individuals included in the study is shown in Table 1.

**Table 1:** Characteristic of lung cancer cases and controls.

Characteristic	Cases (n = 366)	Controls (n = 366)	p-value ^c)^
**Mean year of birth (range)**	1950 (1930–1981)	1950 (1930–1982)	0.96
**Sex, n (%)**			
**M (%)**	230 (62.8%)	230 (62.8%)	-
**F (%)**	136 (37.2%)	136 (37.2%)	-
**Smoking**			
**No ^a)^ (%)**	136 (37.2%)	141 (38.5%)	-
**Yes^b)^ (%)**	230 (63.8%)	225 (61.5%)	-
**Pack-years (range)**	21.93 (0–80)	19.67 (0–72)	0.10
**Histology, n (%)**			
Squamous cell carcinoma	153 (41)	-	-
Adenocarcinoma	145 (38)	-	-
Small cell carcinoma	30 (9)	-	-
Large cell carcinoma	10 (3)	-	-
Other	20 (7)	-	-
Missing	6 (2)	-	-
**Mean folate concentration in nmol/l (range)**	20.07(2.19–57.35)	22.52 (2.18–61.24)	0.002

^a^ former and never smokers

^b^ current smokers

^**c**^ p-value obtained using Mann-Whitney test

All participants were fasting at least six hours before blood samples collection. The study was approved by the Ethics Committee of the Pomeranian Medical University in Szczecin, Poland and all participants gave informed written consent.

### Determination of folate concentration

Quantitative analysis of folate (5-metylotetrahydrofolate) was performed by High Performance Liquid Chromatography (HPLC) method using Flexar HPLC system (PerkinElmer). Analysis was conducted on Macherey-Nagel C18 aquos (250mm x 4,6mm) column, the mobile phase was methanol and 0.6% acetic acid in proportion 14:86 (v/v) with 1ml/min flow rate. The excitation and emission wavelength were 295 and 360 nm, respectively.

Calibration curves were created using 5 calibration standards (50 nmol/l, 80 nmol/l, 100 nmol/l, 120 nmol/l, 150 nmol/l) prepared by dilution of 5-MTHF with 1% ascorbic acid and 0,2M phosphate buffer (K_2_HPO_4_). The calibration curves had a linear character (y = ax + b). Coefficient of determination (R^2^) of linear regression was maintained at least at 0,95. The Relative Standard Deviation (RSD) for folate measurements was 1,55%.

From each study participant 4 ml of venous blood were collected in the vacutainer tubes containing coagulant. The blood was left for 10 minutes at room temperature to form a clot and subsequently centrifuged (3500RPM, 10 minutes) to separate the serum from the clot. The serum was carefully transferred to new labeled sterile Eppendorf tube and stored at -80°C for further analysis, no longer than 4 weeks. According to available literature data, storage at -70°C is sufficient to maintain the original concentration of folate for 1 year [28]. However, we determined experimentally that concentration of folate falls down about 40% in serum samples stored in -80°C longer than 4 weeks.

Before measurement of folate, the serum samples were transferred from the freezer -80°C to room temperature and left to thaw slowly. Afterward, 250μl of serum was transferred to the eppendorf vials containing 150μl of 1% ascorbic acid and 100μl of 1000 nmol/l 5-MTHF standard. After 10 minutes of shaking, 500μl K_2_HPO_4_ with 30 mmol/l mercaptoethanol was added, and samples were incubated in 95°C for 10 minutes to denature serum proteins. Then the samples were centrifuged and supernatant was used directly for HPLC analysis.

Concentration of folate was calculated based on sample peak area and calibration curves and given in nmol/l. Samples from lung cancer cases and controls were measured randomly and the lab stuff was blinded to case-control status.

### Molecular analyses

Six selected variants in 3 genes were genotyped: rs1801133 and rs1801131 in *MTHFR*, rs1805087 in *MTR*, rs1801394, rs1532268 and rs10380 in *MTRR*. All selected SNPs were functional and suggested to be associated with cancer risk including lung cancer [22–27]. From each individual included in the study, a 10ml peripheral blood was collected in a vacutainer tube containing 1 ml of 10% sodium EDTA. The genomic DNA was isolated using detergent method [29]. Analyses were performed using pre-designed Genotyping Assay x 40 (Applied Biosystems). Each reaction mixture consisted of 2,5μl LightCycler 480 Probes Master Mix (Roche Diagnostics), the assay 0,125μl (Genotyping Assay x 40 TaqMan, Applied Biosystems) and 1,375μl deionized water (Roche Diagnostics). Samples were analyzed on 384-well plates. On each plate was included positive, negative and water-blind control. The genotyping data were collected and analyzed using the LightCycler 480 Instrument and the program of the LightCycler 480 Basic Software Version 1.5 (Roche Diagnostics).

### Statistical analysis

The comparison of year of birth, pack-years and folate concentration between cases and controls was performed using Mann-Whitney test. For estimation of association of folate concentration with cancer occurrence, individuals were assigned to one of four categories (quartiles) based on the distribution in the entire group. The association of folate concentration with lung cancer incidence was estimated with odds ratios (OR) at 95% confidence intervals using univariable conditional logistic regression. The quartile with lowest concentration of folate was considered as the ‘reference’ for the calculation of odd ratios (OR).

In addition, OR values where associated with serum folate concentration levels using a sliding window approach. The samples were sorted according to folate concentration and OR values were calculated using the sliding window (size 30 samples) operation. The window was sliding one sample per step over the entire range of serum folate concentration levels. The calculated OR values where then displayed graphically. Furthermore, a local weighted regression (Lowess-regression) was applied on the evaluated OR values, resulting in an estimation of lung cancer incidence curve. Calculations where made using MATLAB.

The association of each of the six tested SNPs (rs1801131 and rs1801133 in MTHFR, rs1805087 in MTR and rs1801394, rs1532268, rs10380 in MTRR) was analyzed by univariable conditional logistic regression. The odds ratios (ORs) with their corresponding 95% CIs were generated using the most common homozygote genotype as the reference group.

In addition, a multivariable conditional logistic regression analysis was performed to evaluate association between circulating folate and each tested SNPs. The aim of this analysis is to distinguish the bare effect of a particular SNP after elimination of potential confounding factors, like other SNPs and folate concentration. Analyses were performed in R (version 3.0.1).

## Results

The lung cancer cases and healthy controls were well matched with respect to year of birth, age, sex and lifetime tobacco exposure (pack-years) (Table 1). The mean concentration of folate was similar in men (21.24 nmol/l) and women (21.39 nmol/l). The folate concentration was lower in smokers (current or past) (20.17nmol/l) than among non-smokers (21.66 nmol/l) although the difference was not statistically significant (p = 0.62). The mean concentration of folate in lung cancer cases was significantly lower than in controls (20.07 nmol/l vs. 22.34 nmol/l, p = 0.002) (Table 1). Analysis in quartiles revealed that the odds of lung cancer decreased with increasing folate concentration (Table 2). The odds ratio for lung cancer cases was significantly lower (OR_uni_ 0.61, 95% CI 0.40–0.95, p = 0.03; OR_multi_ 0.60, 95% CI 0.38–0.94, p = 0.03) in the highest quartile (>27.71nmol/l) in comparison to the lowest (<15.92 nmol/l) quartile in univariable and multivariable (adjusted for six tested SNPs) analysis (Table 2). Similar correlation was observed in analysis of subgroups based on smoking status (OR_uni_ 0.59, p = 0.07; OR_multi_0.56, p = 0.06 in smokers and OR_uni_0.59, p = 0.15; OR_multi_ 0.54, p = 0.11 in non-smokers) and histology (squamous cell carcinoma: OR_uni_ 0.47, 95% CI 0.24–0.93, p = 0.03; OR_multi_ 0.47, 95% CI 0.23–0.98, p = 0.04 and adenocarcinoma: OR_uni_0.63, 95% CI 0.30–1.30, p = 0.21; OR_multi_ 0.59, 95% CI 0.28–1.26, p = 0.21), however only results in subgroup of patients with squamous cell carcinoma were statistically significant (S1 Table).

**Table 2:** Folate concentration and lung cancer incidence.

Quartile	Folate concentration (nmol/l)	Cases n = 366 (%)	Controls n = 366 (%)	OR_uni_ (95%CI)	P-value*	OR_multi_ (95%CI)	P-value**
**I**	2.18–15.92	100 (27.3)	83 (22.7)	1	-	1	-
**II**	16.11–20.20	100 (27.3)	83 (22.7)	0.99 (0.63–1.54)	0.96	1.00 (0.63–1.58)	1.00
**III**	20.22–25.60	86 (23.5)	97 (26.5)	0.70 (0.45–1.09)	0.12	0.69 (0.44–1.08)	0.10
**IV**	25.71–61.24	80 (21.9)	103 (28.1)	0.61 (0.40–0.95)	0.03	0.60 (0.38–0.94)	0.03

* univariable conditional logistic regression

** multivariable conditional logistic regression (folate concentration adjusted for six tested SNP’s)

**Fig 1:**
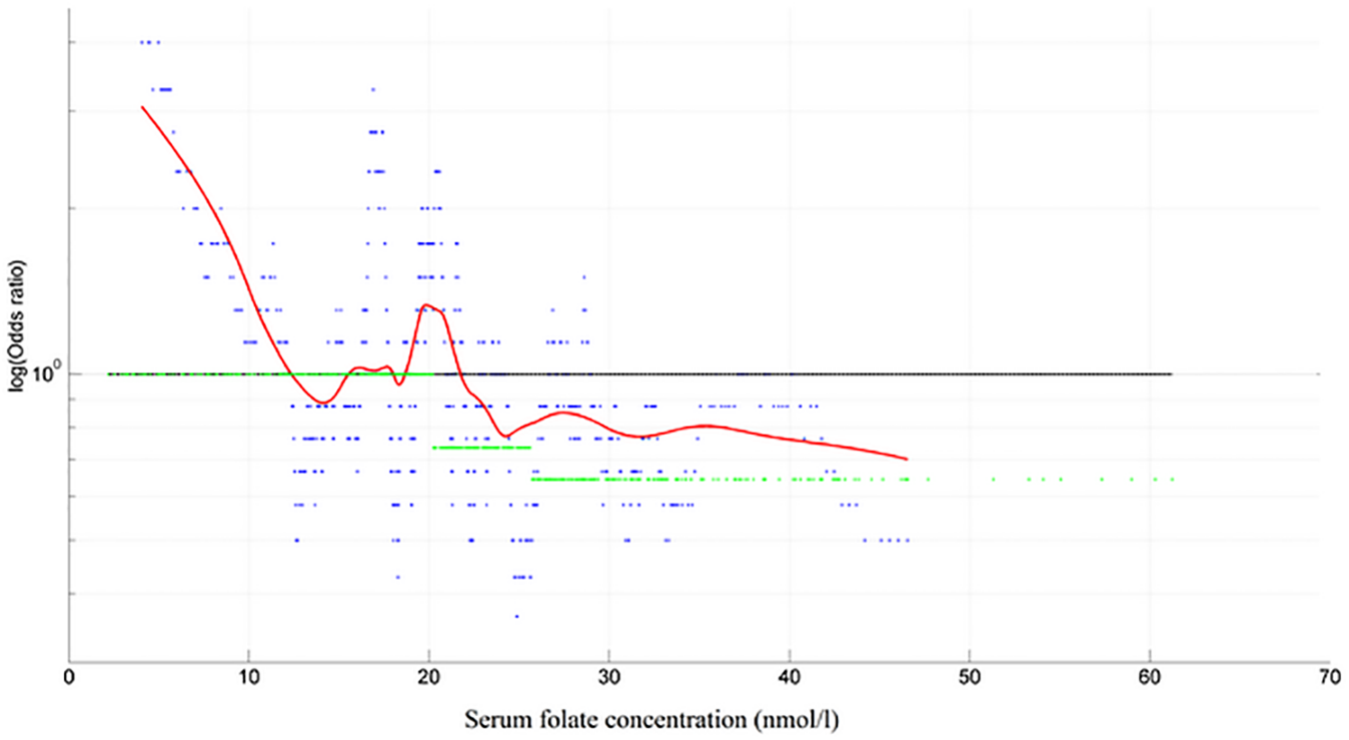
Serum folate concentration and odds ratio curve of developing lung cancer. Odds ratio curve of developing lung cancer for folate concentration (nmol/l) shown for a sliding approach (window size: 30 observations). Values above the line indicate an increased probability, values below indicate a decreased probability of developing lung cancer. The reference proportions is 1:1 for the entire series. A Lowest-regression was applied to those calculated data point to estimate the underlying probability curve.

Analysis of the estimated OR curve (sliding window analysis) shown that odds of developing lung cancer substantially increases for folate concentration <12 nmol/l, but decreases for concentration >22nmol/l (Fig 1).

The distribution of the genotypes in six tested SNPs in *MTHFR* (rs1801131, rs1801133), *MTR* (rs1805087) and *MTRR* (rs1801394, rs1532268, rs10380) genes was not significantly different between lung cancer patients and healthy controls (S2 Table). Analysis of association of these variation with lung cancer risk did not reveal any significant correlation in univariable and multivariable (confounding factors included: folate concentration and SNPs) conditional logistic regression analysis (all data are presented in S2 Table).

## Discussion

In this study, we analyzed serum folate concentration and six variants in three genes associated with folate metabolism in a group of matched lung cancer cases and healthy controls. We found that serum folate level was lower in cancer cases than in healthy controls and was associated with about 40% reduced odds of lung cancer diagnosis (OR_multi_ 0.60, 95% CI 0.38–0.94) in individual with folate concentration >25.71nmol/l in comparison to <15.92 nmol/l.

An association of folate concentration with lung cancer risk was analyzed in a number of case-control studies, however results were inconsistent. These studies differed between in terms of study design (retrospective [8–11] or prospective [12–18]) and/or data analyses (based on dietary questionnaires [8,11,12,15–18] or determination of serum/plasma folate concentration [9,10,13,14]). Significant association of higher folate intake with lower lung cancer risk was reported in 2 case-control [8, 12] and 2 observational cohort studies [11, 15]. However, this relationship was not confirmed in other analyses [16–18]. Several studies examined an association of folate concentration with lung cancer but results were inconclusive. Significantly lower folate concentration was detected in cancer patients in a small retrospective study of 40 cases and 40 controls [9]. However, no difference in folate concentration was found between cases and controls in another retrospective study of similar size (46 cases and 44 controls) [10]. In a nested case-control study conducted within the Alpha-Tocopherol, Beta-Carotene Cancer Prevention Study cohort no significant association was seen between serum folate and lung cancer risk in 300 cases and 300 controls [14]. However, it should be emphasized that in this trial participants received alpha-tocopherol or beta-carotene or placebo, so the results could be influenced by these supplements. A significantly lower risk of lung cancer associated with elevated serum folate was reported in a large study of 899 lung cancer cases and 1815 controls from EPIC cohort [13]. These authors observed a moderate lower risk for increasing serum folate levels, although this association was restricted to former and current smokers and was not apparent in never smokers [13]. In our study we did not detect any statistically significant difference in subgroups depending on smoking, although folate concentration was lower in smokers than in non-smokers. In meta-analysis of 4 case-control studies and 44 cases out of a cohort of 1988 participants a marginal association without significance between high serum folate levels and lower lung cancer susceptibility was reported. Exclusion of single study in the sensitivity test exerted a significant inverse relationship between serum folate levels and lung cancer risk [19]. The observed discrepancies in results between studies may be explained by differences in their size or design, and baseline folate concentration. Results of our retrospective study suggest that serum folate concentration is inversely associated with lung cancer diagnosis. This is consistent with the EPIC cohort, one retrospective study and meta-analysis [9, 13, 19]. The mean folate concentration in our study was 22.52 nmol/l in controls, what is higher in comparison to that reported in all other analyses (14.4 nmol/l in EPIC [13], 4.3 ng/ml (9.7 nmol/l) in Alpha-Tocopherol, Beta-Carotene Cancer Prevention Study [14], and 6.1 pg/ml [9] and 7.8 ng/ml (17.7 nmol/l) [10] in retrospective studies). It is not clear how to explain this variation in folate concentration in particular studies/populations. It could be caused by mandatory fortification in some countries, but also might be related to different methods used for folate determination, as well as processing, shipping, and storage of biological samples. In previously published analyses for determination of folate concentration were used microbiological or protein-binding methods [9, 10, 13, 14]. It is unlikely, but in the microbiological assay antibiotics and antifolates present in the tested sample may potentially influence grow of the bacteria, and therefore cause false results [30]. In the protein-binding assay the binding affinity is influenced by several factors (e.g. pH or protein content in the sample) which might distort the result [30]. In our analysis, for determination of folate we used high-performance liquid chromatography (HPLC). The HPLC method offers the possibility of measuring individual folate forms and is more accurate than the previously used methods [31]. It is known that folate and its metabolites are sensitive to external factors and thermal storage conditions [28, 32]. In our study all serum samples from 366 lung cancer cases and 366 controls were stored in -80°C no longer than 4 weeks before folate determination, so we believe that our results are not confounded by any laboratory factors.

Folate metabolism depends on a number of enzymes that are functionally polymorphic. It has been suggested that the lung cancer risk can be associated with polymorphisms in *MTHFR*, *MTR* and *MTRR*, and this association may be modulated by folate intake [26, 27]. In this study we genotyped 6 variations in 3 genes related to folate metabolism: rs1801133 and rs1801131 in *MTHFR*, rs1805087 in *MTR*, rs1801394, rs1532268 and rs10380 in *MTRR*. We did not found any significant difference between lung cancer cases and controls in univariable and multivariable analyses in which were considered folate concentration and other SNPs as confounders. This potentially may suggest that tested SNPs are not associated with lung cancer risk. However, we can not exclude that the sample size in our study was too small to detect significant effect. Further studies on larger groups are necessary to investigate possible interaction of these SNPs with folate concentration.

There are limitations of the case-control approach. The serum samples for determination of folate concentration in cases were collected at the time of lung cancer diagnosis but before therapy, so it is possible that the folate level could be influenced by the presence of cancer. In this case control study we are not able to determine if folate is a marker of lung cancer diagnosis or a contributing etiologic factor.

An advantage of our study was matching of cases and controls with respect to year of birth, sex, total number of lung and other cancers among first degree relatives and smoking. Therefore, it is very unlikely that the detected association could be confounded by those factors, but it is possible that another confounding variable could introduce bias. In respect of smoking, we matched lung cancer cases and controls only for lifetime tobacco exposure (pack-years) and did not consider time of smoking/quitting. Therefore, the calculated ORs could be stronger than if we account for the massive over risk associated with smoking intensity in current smokers and time since quitting in former smokers. An advantage of our study was application of rigorous conditions of storage and preparation of serum samples for determination of folate concentration. It is known that folate is very unstable and inappropriate handling of biological samples can cause a significant decrease of folate level [28, 32].

## Conclusion

In this case-control study, lower serum folate concentrations were associated with a higher risk of lung cancer diagnosis. Although previous findings have been somewhat mixed, our results add to the evidence that circulating folate levels may be an indicator of lung cancer risk.

## Supporting information

S1 Table Folate concentration and lung cancer incidence in subgroups depending on smoking status and histology. (DOCX)

S2 Table Analysis of variations in *MTHFR*, *MTR* and *MTRR* genes and lung cancer risk. (DOCX)
