# Supplementary material for: Serum folate concentration and the incidence of lung cancer
Source: PLoS One. 2017 May 11;12(5):e0177441. doi: 10.1371/journal.pone.0177441 (PMC5426769; doi:10.1371/journal.pone.0177441)
Supplement: S1 Table — (DOCX) [file pone.0177441.s001.docx]

**S1 Table. Folate concentration and lung cancer incidence in subgroups depending on smoking status and histology.**

| **Quartile** | **Folate concentration (nmol/l)** | **Cases n (%)** | **Controls n (%)** | **OR_uni_ (95%CI) *** | **P-value** | **OR_multi_ (95%CI) **** | **P-value** |
| --- | --- | --- | --- | --- | --- | --- | --- |
| Smoking | | | | | | | |
| **I** | 2.18 - 16.15 | 63 | 51 | 1 | - | 1 | - |
| **II** | 16.16 - 20.16 | 67 | 47 | 1.21 (0.69-2.14) | 0.50 | 1.22 (0.67-2.21) | 0.52 |
| **III** | 20.17 - 25.46 | 50 | 64 | 0.58 (0.32-1.04) | 0.07 | 0.56 (0.31-1.03) | 0.06 |
| **IV** | 25.47 - 61.24 | 50 | 64 | 0.58 (0.32-1.04) | 0.07 | 0.56 (0.31-1.02) | 0.06 |
| Nonsmoking | | | | | | | |
| **I** | 2.19 - 16.04 | 37 | 32 | 1 | - | 1 | - |
| **II** | 16.05 - 20.24 | 33 | 36 | 0.66 (0.30-1.41) | 0.28 | 0.61 (0.26-1.39) | 0.24 |
| **III** | 20.25 - 25.83 | 36 | 33 | 0.92 (0.44-1.92) | 0.82 | 0.88 (0.41-1.87) | 0.74 |
| **IV** | 25.83 - 58.98 | 30 | 39 | 0.59 (0.29-1.22) | 0.15 | 0.54 (0.25-1.16) | 0.11 |
| Adenocarcinoma | | | | | | | |
| **I** | 2.19 - 16.24 | 38 | 35 | 1 | - | 1 | - |
| **II** | 16.25 - 19.80 | 39 | 33 | 1.07 (0.53-2.15) | 0.85 | 1.06 (0.51-2.22) | 0.88 |
| **III** | 19.81 - 24.39 | 37 | 35 | 0.96 (0.49-1.90) | 0.92 | 1.00 (0.49-2.02) | 1.00 |
| **IV** | 24.40 - 46.40 | 31 | 42 | 0.63 (0.30-1.30) | 0.21 | 0.59 (0.28-1.26) | 0.21 |
| Squamous cell carcinoma | | | | | | | |
| **I** | 2.18 - 15.60 | 44 | 33 | 1 | - | 1 | - |
| **II** | 15.61 - 20.25 | 42 | 34 | 0.88 (0.44-1.79) | 0.73 | 0.91 (0.43-1.93) | 0.81 |
| **III** | 20.25 - 26.20 | 36 | 40 | 0.59 (0.28-1.24) | 0.16 | 0.57 (0.26-1.26) | 0.17 |
| **IV** | 26.21 - 58.98 | 31 | 46 | 0.47 (0.24-0.93) | 0.03 | 0.47 (0.23-0.98) | 0.04 |

* univariable conditional logistic regression

** multivariable conditional logistic regression (as described in the Statistical Analysis)
