# Supplementary material for: Serum folate concentration and the incidence of lung cancer
Source: PLoS One. 2017 May 11;12(5):e0177441. doi: 10.1371/journal.pone.0177441 (PMC5426769; doi:10.1371/journal.pone.0177441)
Supplement: S2 Table — (DOCX) [file pone.0177441.s002.docx]

**S2 Table . Analysis of variations in *MTHFR*, *MTR* and *MTRR* genes and lung cancer risk.**

| Genotypes | Cases, n=366 (%) | Controls, n=366 (%) | OR_uni_* | 95%CI | p-value | OR_multi_** | 95%CI | p-value |
| --- | --- | --- | --- | --- | --- | --- | --- | --- |
| rs1801131 in *MTHFR* | | | | | | | | |
| AA | 178 (49) | 183 (50) | 1 | - | - | 1 | - | - |
| AC | 151 (41) | 150 (41) | 1.04 | 0.76 - 1.41 | 0.81 | 1.07 | 0.78 - 1.46 | 0.67 |
| CC | 37 (10) | 33 (9) | 1.16 | 0.69 - 1.93 | 0.58 | 1.17 | 0.70 - 1.98 | 0.55 |
| rs1801133 in *MTHFR* | | | | | | | | |
| CC | 169 (46) | 154 (42) | 1 | - | - | 1 | - | - |
| CT | 160 (44) | 169 (46) | 0.86 | 0.63 - 1.18 | 0.34 | 0.85 | 0.62 - 1.17 | 0.32 |
| TT | 37 (10) | 43 (12) | 0.80 | 0.50 - 1.27 | 0.34 | 0.77 | 0.48 - 1.23 | 0.28 |
| rs1805087 in *MTR* | | | | | | | | |
| AA | 190 (52) | 197 (54) | 1 | - | - | 1 | - | - |
| AG | 144 (39) | 128 (35) | 1.18 | 0.85 - 1.65 | 0.32 | 1.23 | 0.88 - 1.73 | 0.22 |
| GG | 32 (9) | 41 (11) | 0.83 | 0.50 - 1.37 | 0.46 | 0.86 | 0.52 - 1.43 | 0.57 |
| rs1801394 in *MTRR* | | | | | | | | |
| AA | 119 (33) | 122 (34) | 1 | - | - | 1 | - | - |
| AG | 183 (50) | 182 (50) | 1.03 | 0.75 - 1.42 | 0.86 | 1.00 | 0.72 - 1.39 | 0.99 |
| GG | 64 (17) | 62 (16) | 1.06 | 0.69 - 1.63 | 0.80 | 1.05 | 0.68 - 1.63 | 0.83 |
| rs1532268 in *MTRR* | | | | | | | | |
| GG | 160 (44) | 158 (43) | 1 | - | - | 1 | - | - |
| GA | 166 (45) | 164 (45) | 1.00 | 0.73 - 1.36 | 0.99 | 1.00 | 0.73 - 1.37 | 0.98 |
| AA | 40 (11) | 44 (12) | 0.89 | 0.54 - 1.47 | 0.64 | 0.87 | 0.52 - 1.45 | 0.59 |
| rs10380 in *MTRR* | | | | | | | | |
| CC | 338 (92) | 329 (90) | 1 | - | - | 1 | - | - |
| CT+TT^#^ | 28 (8) | 37 (10) | 0.75 | 0.46 - 1.24 | 0.26 | 0.74 | 0.45 - 1.23 | 0.25 |

* univariable conditional logistic regression

** multivariable conditional logistic regression (as described in the Statistical Analysis)

^#^one lung cancer patient had TT genotype
